# Supplementary material for: Crawling and Gliding: A Computational Model for Shape-Driven Cell Migration
Source: PLoS Comput Biol. 2015 Oct 21;11(10):e1004280. doi: 10.1371/journal.pcbi.1004280 (PMC4619082; doi:10.1371/journal.pcbi.1004280)
Supplement: S1 Code — (ZIP) [file pcbi.1004280.s012.zip › release/tst/doc/html/classDir-members.html]

Tissue Simulation Toolkit: Member List


Tissue Simulation Toolkit
 0.1.4.1


- Main Page
- Namespaces
- Classes
- Files

- Class List
- Class Hierarchy
- Class Members

Dir Member List

This is the complete list of members for Dir, including all inherited members.

|  |  |  |
| --- | --- | --- |
| aa1 | Dir |  |
| aa2 | Dir |  |
| bb1 | Dir |  |
| bb2 | Dir |  |
| CellularPotts class | Dir | friend |
| Dir() | Dir | inline |
| lb1 | Dir |  |
| lb2 | Dir |  |


---

Generated on Thu Aug 14 2014 22:04:01 for Tissue Simulation Toolkit by  

 1.8.6
